# A global distributed basin morphometric dataset

**DOI:** 10.1038/sdata.2016.124

**Published:** 2017-01-05

**Authors:** Xinyi Shen, Emmanouil N. Anagnostou, Yiwen Mei, Yang Hong

**Affiliations:** 1 Department of Civil & Environmental Engineering, University of Connecticut, Storrs, Connecticut 06269-3037, USA; 2 Advanced Radar Research Center, National Weather Center, University of Oklahoma, Norman, Oklahoma 73072, USA

**Keywords:** Hydrology, Natural hazards, Climate change

## Abstract

Basin morphometry is vital information for relating storms to hydrologic hazards, such as landslides and floods. In this paper we present the first comprehensive global dataset of distributed basin morphometry at 30 arc seconds resolution. The dataset includes nine prime morphometric variables; in addition we present formulas for generating twenty-one additional morphometric variables based on combination of the prime variables. The dataset can aid different applications including studies of land-atmosphere interaction, and modelling of floods and droughts for sustainable water management. The validity of the dataset has been consolidated by successfully repeating the Hack’s law.

## Background & Summary

Morphometry, the topographic and bathymetric features of the earth surface, is known as interactions among multiple factors including climate, tectonic, and erosion, and is known to impact landscape, ecology, and consequentially the occurrence and severity of hydro-meteorological hazards. To understand how the natural surface has grown into its current state^1–4^, what it will become^5^, and in which way it impacts the environment^6–12^, we need distributed geomorphological data at global scale. The most commonly cited geomorphological features, listed in Table 1, were defined nearly 20 years ago, while currently a number of global or regional gridded topographic datasets^13–16^ are available to support newly derived geomorphological features.

Numerous local geomorphological studies have been conducted using sparse and limited data^4,6,8,9,17,18^. Only uniform geomorphological features are available for large basins^12,19^. Due to the heavy computation of basin delineation and boundary tracing at global scale, some critical features missing from existing datasets are based on boundary information such as basin length and perimeter. A common solution has been to convert those features from easy-to-obtain features (such as drainage area) by means of statistical relations^20^, which is bound to empirical experience and less accuracy, as will be shown in the Technical Validation Section.

The objective of this paper is to share the first distributed global geomorphological dataset available at 30 arc seconds (denoted as 30’ hereafter) resolution. This dataset groups 30 basin characteristics into two categories, prime (the first 9 variables) and derived (the rest 21 variables) as listed in Table 1. The prime characteristic variables are computed strictly by geomorphic definitions following the from-upstream-to-downstream (FUTD) framework^21^ and using all cells within the basin, while the derived variables are calculated numerically based on the prime variables, therefore they are not archived.

## Methods

The dataset is made available by a recently released tool^21^ that can reduce the computation to linear complexity, O(*N*). Input data used in the morphometric characteristics’ computations include digital elevation model (DEM) flow direction (FDR) and flow accumulation (FAC) maps at 30’ resolution contained in the global shuttle elevation derivatives available at multiple Scales (HydroSHEDS) dataset. The tool is built on a FUTD framework that starts from the most upstream grids (where FAC is equal to 1) and then ‘flows’ to the downstream direction while computing. Redundant computations are avoided by inheriting tributary basin characteristics and eliminating the process of basin delineation and boundary tracing. Through this process, each grid is visited minimal times, which maximizes computation efficiency. For the details of calculating each prime variable in the FUTD framework, a demonstration of the algorithm for a small-scale basin consisting of 44 grids is given at this product’s website, http://engr.uconn.edu/~xshen/GDBC/#example.

### Code availability

The matlab codes and user manual of the tool used to generating the dataset are accessible at http://engr.uconn.edu/~xshen/GDBC/software/.

## Data Records

**Figure 1:**
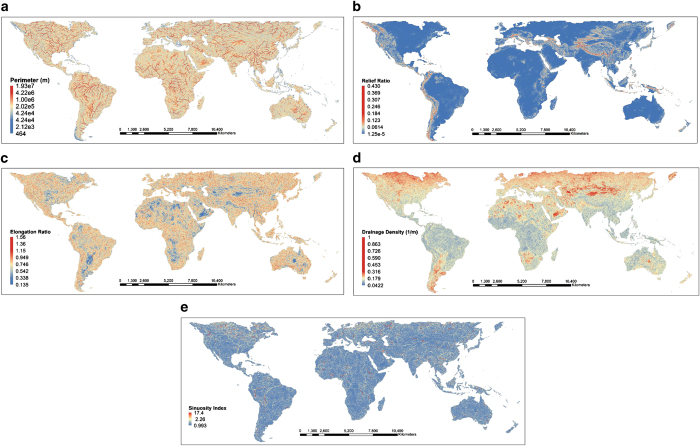
Selected Geomorphological Variables of the proposed dataset: (**a**) perimeter, (**b**) relief ratio, (**c**) elongation ratio, (**d**) drainage density and (**e**) sinuosity.

**Figure 2:**
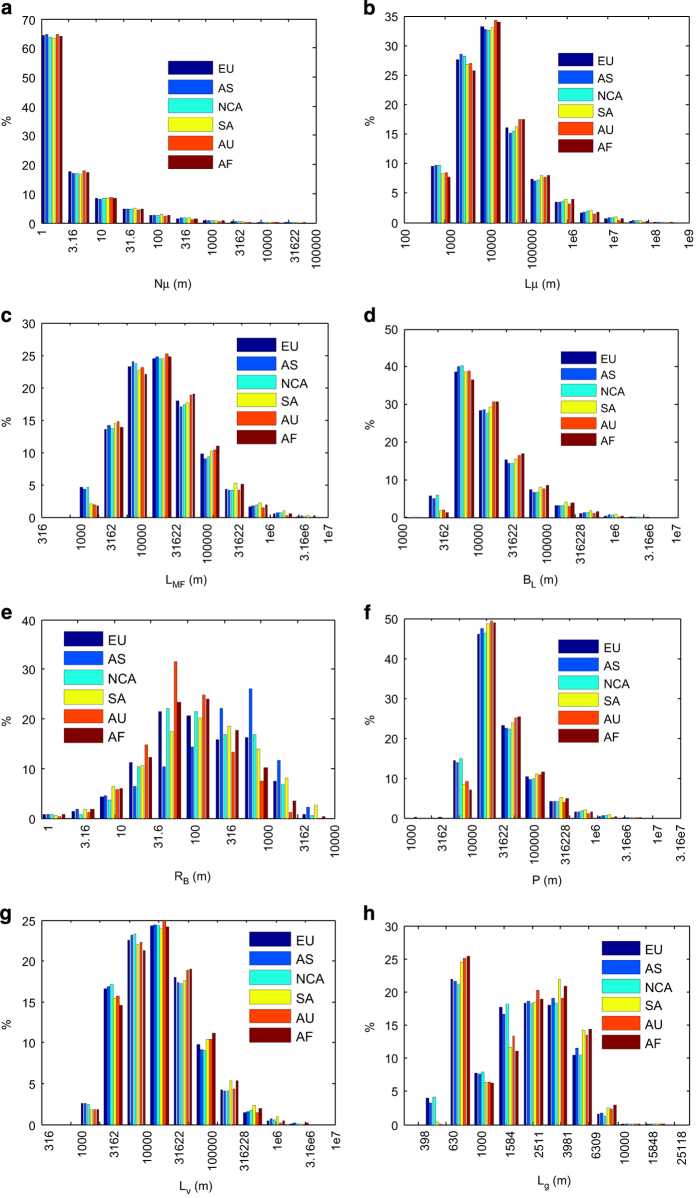
Distribution of prime basin characteristics: (**a**) **Nμ** (**b**) **Lμ**, (**c**) **L**_**MF**_, (**d**) **L**_**B**_, (**e**) **R**_**B**_, (**f**) **P**, (**g**) **L**_**v**_, and (**h**) **L**_**g**_, grouped by continent: Europe (EU), Asia (AS), North and Central America (NCA), South America (SA), Australia (AU) and Africa (AF). **N**_**μ**_ and **L**_**μ**_ are only displayed for first order streams, i.e., μ=1.

The HydroSHEDS dataset^13^ used in this study can be accessed at http://www.hydrosheds.org. Figure 1 gives snapshot of some selected basin characteristics. In Fig. 1b, large relief ratio appears at mountainous areas including the Alps-Himalaya belt, Cordillera belt, Altai belt, and New Guinea highlands. The probability of basins with high drainage density roughly increases with latitude in both hemispheres. Figure 2 shows the distribution (converted from number of grids to percentage) of prime variables grouped by continent. It shows that distributions of any given prime variable except the basin relief are almost identical among different continents. The significant distinction between basin relief and other prime variables is that the former is a vertical measurement while the latter are all horizontal descriptors.

The nine prime variables are can be accessed at figshare via https://figshare.com/s/6cd00491b850bad716d7 (Data Citation 1). Files are stored in GeoTiff format and are projected in world geodetic system 1984 (WGS84). Basin characteristics are compressed into a single file for each continent. An example file name is ‘AF.zip’ with AF standing for Africa. The rest continents are AS for Asia, EU for Europe, AU for Australia, CA for Central America, NA for North America and SA for South America. One will find the file, ‘AF_BL.tif’, among other characteristics by decompressing the ‘AF.zip’ file. Other variable abbreviations include BR, Lg, Nu, Lu, Lv, MFL, P and SO, standing for basin relief, length of overland flow, stream number, stream length, down valley length, main (maximal) flow length, perimeter and stream order, respectively. It is noted that each variable appears stored in a single band image file except stream number and stream length that are stored in stream order-indexed multi-band files. Therefore, the number of bands of stream number and stream length files depends on the maximum stream order for a given continent.

## Technical Validation

### Quality control of the production method

The production method is carried out using the recently published algorithm^21^, with every variable strictly following their original definition listed in Table 1. The algorithm is fully automated, therefore main error sources include errors in the input data i.e., the HydroSHEDS dataset, and on the assumption of single flow direction (SFD). The first error source is primarily due to the existence of dense vegetation, unknown situation under permanent water and the upscaling process, however can be mitigated by a proposed procedure of correcting the dataset^22^. The effect of the second error is mitigated using 30’ resolution.

### Validation using Hack’s law and closing remarks

Since similar datasets do not exist for comparison, we performed indirect validation of the proposed dataset via the Hack’s law.The Hack’s law is an empirical power law between drainage area, ***A*** and different measures of length, ***L***, main flow or basin length, as written in equation (1), which was originally proposed by fixing ***C*** and ***n*** to 1.4 and 0.6 respectively^20^, the modified by^23,24^ to improve the estimation of ***n***, and finally generalized as cumulative density function for both basin area and length, as given by equations (2) and (3), most recently^25–27^.

(1)L=CAn

(2)P(A>a)∝a−β

(3)P(LB>l)∝l−β/n≡l−γ

where

(4)β=1−n

Using the proposed dataset, we first tested the accuracy of equation (1) by regressing ***C*** and ***n*** for all grids in each continent, then that of equations (2) and (3) in the long river in each continent. From equation (2) the probability density function (PDF) of drainage area can be written by equation (5):

(5)p(A)∝A−(1+β)

If we set

(6)B=ln(A)

then,

(7)p(B)∝e−βB

Similarly,

(8)p(M)∝e−γM

where,

(9)M=ln(LB)

The distribution of ***B*** and ***M*** are easier to be visualized than A and ***L***_***B***_ because the high concentration on basins of small scales. It is understood that grids of ***L***_***B***_<10 km are ruled out for this validation because of the possibility of losing accuracy of small ***L***_***B***_ derived from 1 km source data. Following the convention of the Hack’s law, the unit of length and area are converted to mile and squared mile before fitting. Since the method of computing***n***remains controversial in the past literatures, one way to validate equations (7) and (8) is through inspecting the linearity of ***ln*** [***p*** (***B***)] and ***ln*** [***p*** (***M***)].

**Figure 3:**
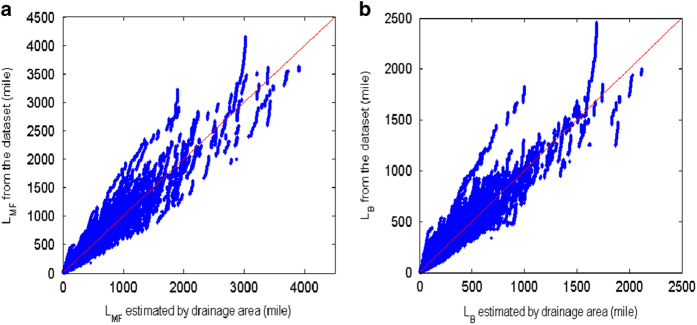
Validation of the Hack’s law: (**a**) basin area versus main flow length and (**b**) basin area versus basin length. The correlation value and RMSE of this fitting are given in Table 2.

**Figure 4:**
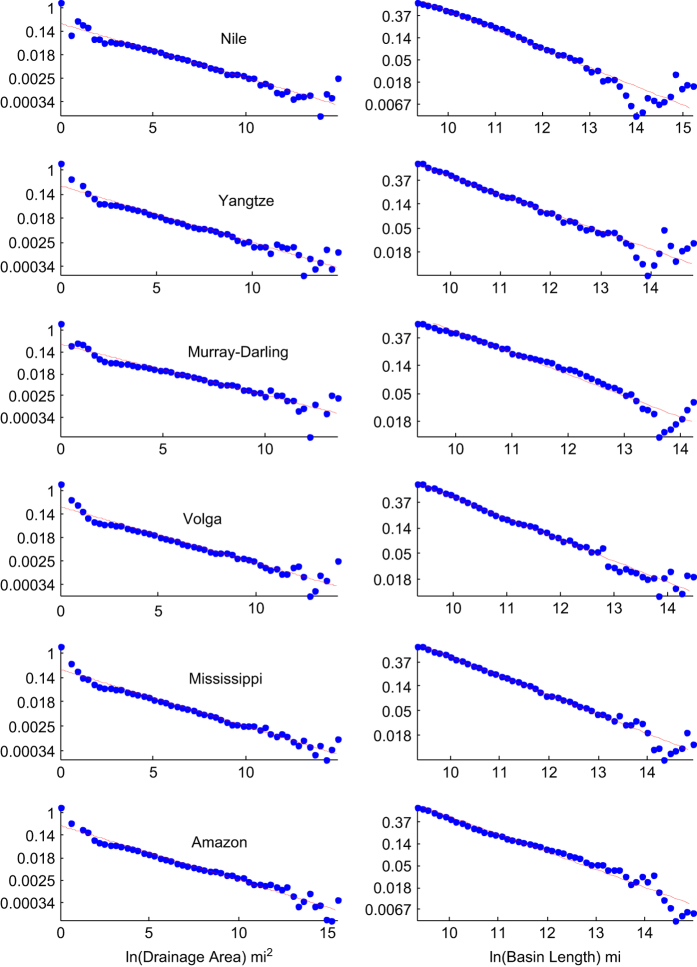
Validation of the PDF of drainage area and basin length given by equations (7) and (8) in the left and right column respectively. Each row contains the results from a river. From the top to the bottom, they are Nile River in Africa, Yangtze River in Asia, Murray–Darling River in Australia, Volga River in Europe, Mississippi River in North America, and Amazon River in South America.

Scatter plots of equation (1) with setting ***L*** to ***L***_***MF***_ and ***L***_***B***_ are given in Fig. 3a,b. The Pearson correlation coefficient varies from 0.96 to 0.99 and the root mean squared error (RMSE) varies from 10.95 to 41.67 mi for ***L***_***MF***_ and from 4.982 to 12.00 mi for ***L***_***B***_, respectively, as given in Table 2. The linearity of the pdf of equations (7) and (8) are tested in the following river basins, Nile, Yangtze, Mississippi, Amazon, Murray-Darling and Volga Rivers, as shown in Fig. 4. The goodness of fit of the distribution and the estimated ***β*** and ***γ*** are listed in Table 3. Except the slight deviation at both ends, the overall power law distribution is very well represented by the proposed dataset with obtaining Pearson correlation coefficients from 0.89–0.98, and the estimated ***β*** fallen between 0.4–0.5 (indicating that ***n*** is between 0.5–0.6). At this point, we have proved that the proposed data satisfy the Hack’s law.

## Additional information

**How to cite this article**: Shen, X. *et al.* A global distributed basin morphometric dataset. *Sci. Data* 4:160124 doi: 10.1038/sdata.2016.124 (2017).

**Publisher’s note**: Springer Nature remains neutral with regard to jurisdictional claims in published maps and institutional affiliations.

## Supplementary Material



## Figures and Tables

**Table 1:** Basin characteristics included in the proposed dataset.

**Variable (File Name)**	**Description**	**Definition**	**References**
***S***_***μ***_ **(SO)**	Stream Order(Strahler)	Strahler stream order, numerical measure of river’s branching complexity	^28^
***N***_***μ***_ **(Nu)**	Stream Number	order-wise stream segments based on ***S***_***μ***_	^29^
***L***_***μ***_ **(Lu)**	Stream Length	order-wise total stream length based on ***S***_***μ***_	^29^
***L***_***MF***_ **(MFL)**	Maximal Flow Length	the length along the longest watercourse from the mouth to the head of the channel	^30^
***L***_***v***_ **(Lv)**	Down Valley Length	The straight distance from the river cell of interest to the basin mouth	^30^
***L***_***g***_ **(Lg)**	Length of Overland Flow	The overland flow length to river	^29^
***R***_***B***_ **(BR)**	Basin Relief	The elevation difference between the highest point on the drainage divide and the mouth	^10^
***L***_***B***_ **(BL)**	Basin Length	The maximal length of the line from a basin mouth to a point on the perimeter equidistant from the basin mouth in either direction around the perimeter	^31^
***P*****(P)**	Basin Perimeter	The outer boundary of the watershed that enclosed its area	^32^
***R***_***b***_	Bifurcation Ratio	***R***_***bμ***_=**N**_***μ***_/**N**_***μ*****+1**_ (10)	^32^
***B***_***W***_	Weighted Mean Bifurcation Ratio	$B_{w}=\frac{1}{\sum_{1}^{\max\left(S_{µ}\right)-1}\left(N_{µ}+N_{µ+1}\right)}\sum_{1}^{\max\left(S_{µ}\right)-1}R_{bµ}\left(N_{µ}+N_{µ+1}\right)$ (11)	^33^
***L***_***mμ***_	Mean Stream Length	***L***_***mμ***_=***L***_***μ***_/***N***_***μ***_ (12)	^34^
***L***_***mrμ***_	Stream Length Ratio	***L***_***mrμ***_=***L***_***μ***_/***L***_***μ-1***_ (13)	^29^
***S***_***i***_	Sinuosity Index	***S***_***i***_=***L***_***MF***_/***L***_***v***_ (14)	^35^
***F***_***f***_	Form Factor	***F***_***f***_=***A***/***L***_***B***_, where A is the drainage area (15)	^36^
***R***_***r***_	Relief Ratio	***R***_***r***_***=R***_***B***_***/L***_***B***_ (16)	^32^
***R***_***e***_	Elongation Ratio	***R***_***e***_=***2***/***L***_***B***_***×(A/π)***^***0.5***^ (17)	^32^
***R***_***t***_	Texture Ratio	***R***_***t***_=***N***_***1***_***/P*** (18)	^29^
Rc	Circularity Ratio	***R***_***c***_=***4πA/P***^***2***^ (19)	^37^
***k***	Lemniscate’s value	***k***=***L***_***B***_^***2***^***/A*** (20)	^38^
***D***_***tμ***_	Drainage Texture	***D***_***tμ***_=***N***_***μ***_***/P*** (21)	^29^
***D***_***d***_	Drainage Density	***D***_***d***_***=L***_***μ***_/***A*** (22)	^31,36^
***C***_***c***_	Compactness Coefficient	***C***_***c***_***=***0.2841 ***P/A***^***0.5***^ (23)	^39^
***R***_***W***_	Wandering Ratio	***R***_***W***_=***L***_***MF***_/***L***_***B***_ (24)	^40^
***R***_***f***_	Fitness Ratio	***R***_***f***_=***L***_***MF***_/***P*** (25)	^41^
***M***_***B***_	Basin Magnitude	***M***_***B***_=***N***_**1**_ (26)	^10^
***F***_***s***_	Channel Frequency	***F***_***s***_=***N***_***μ***_***/A*** (27)	^36^
***D***_***i***_	Drainage Intensity	***F***_***s***_/***D***_***d***_ (28)	^42^
***I***_***f***_	Infiltration Number	***I***_***f***_***=F***_***s***_×***D***_***d***_ (29)	^42^
***R***_***n***_	Ruggedness Number	***R***_***n***_***=R***_***B***_×***D***_***d***_ (30)	^43^

**Table 2:** Fitting error and coefficients of the Hack’s law.

	**AF**	**AS**	**AU**	**NA**	**CA**	**SA**	**EU**	**AF**	**AS**	**AU**	**NA**	**CA**	**SA**	**EU**
	Pearson Correlation							RMSE (mi)						
***L***_***MF***_	0.9828	0.9633	0.9754	0.9875	0.9912	0.9798	0.9760	25.60	41.67	15.97	22.11	10.95	31.79	21.23
***L***_***B***_	0.9765	0.9629	0.9680	0.9837	0.9846	0.9782	0.9668	9.695	12.00	6.415	7.520	4.982	10.39	7.840
	C							n						
***L***_***MF***_	1.8102	1.4318	1.8672	1.9455	0.8945	2.8166	2.1845	0.5329	0.5715	0.5268	0.5326	0.6157	0.4944	0.5168
***L***_***B***_	2.0910	1.7300	1.9922	2.0133	0.9584	2.8204	2.3020	0.4808	0.5048	0.4825	0.4829	0.5696	0.4526	0.4641

**Table 3:** Slope and the goodness of-fit of equations (7) and (8).

	**Nile**	**Yangtze**	**Murray-Darling**	**Mississippi**	**Amazon**	**Volga**	**Nile**	**Yangtze**	**Murray-Darling**	**Mississippi**	**Amazon**	**Volga**
Pearson Correlation							Slope (β for ***p***(***B***) and γ for ***p***(***M***))					
***p(B)***	0.9312	0.9346	0.8935	0.9590	0.9568	0.9210	0.4679	0.4667	0.4628	0.4621	0.4853	0.4798
***p***(***M)***	0.9196	0.9345	0.9485	0.9736	0.9594	0.9753	0.8566	0.7536	0.7671	0.7907	0.8080	0.8243

## References

[d1] Figshare Shen X. 2016 http://dx.doi.org/10.6084/m9.figshare.c.3302111

